# Temporal Asynchrony but Not Total Energy Nor Duration Improves the Judgment of Numerosity in Electrotactile Stimulation

**DOI:** 10.3389/fbioe.2020.00555

**Published:** 2020-06-19

**Authors:** Sara Nataletti, Fabrizio Leo, Lucia Seminara, Carlo Trompetto, Maurizio Valle, Strahinja Dosen, Luca Brayda

**Affiliations:** ^1^Robotics, Brain and Cognitive Science Department, Istituto Italiano di Tecnologia, Genoa, Italy; ^2^Department of Informatics Bioengineering Robotics, and System Engineering, University of Genoa, Genoa, Italy; ^3^Department of Electrical, Electronic, Telecommunications Engineering and Naval Architecture, University of Genoa, Genoa, Italy; ^4^Department of Neuroscience, IRCCS Ospedale Policlinico San Martino, Genoa, Italy; ^5^Department of Neuroscience, Rehabilitation, Ophthalmology, Genetics and Maternal and Child Sciences, University of Genoa, Genoa, Italy; ^6^Department of Health Science and Technology, Faculty of Medicine, Aalborg University, Aalborg, Denmark; ^7^Acoesis Inc., Genoa, Italy

**Keywords:** sensory substitution, numerosity judgment, electrotactile stimulation, stimulation timing, tactile feedback, upper extremity, somatosensory integration, touch

## Abstract

Stroke patients suffer from impairments of both motor and somatosensory functions. The functional recovery of upper extremities is one of the primary goals of rehabilitation programs. Additional somatosensory deficits limit sensorimotor function and significantly affect its recovery after the neuromotor injury. Sensory substitution systems, providing tactile feedback, might facilitate manipulation capability, and improve patient's dexterity during grasping movements. As a first step toward this aim, we evaluated the ability of healthy subjects in exploiting electrotactile feedback on the shoulder to determine the number of perceived stimuli in numerosity judgment tasks. During the experiment, we compared four different stimulation patterns (two simultaneous: short and long, intermittent and sequential) differing in total duration, total energy, or temporal synchrony. The experiment confirmed that the subject ability to enumerate electrotactile stimuli decreased with increasing the number of active electrodes. Furthermore, we found that, in electrotactile stimulation, the temporal coding schemes, and not total energy or duration modulated the accuracy in numerosity judgment. More precisely, the sequential condition resulted in significantly better numerosity discrimination than intermittent and simultaneous stimulation. These findings, together with the fact that the shoulder appeared to be a feasible stimulation site to communicate tactile information via electrotactile feedback, can serve as a guide to deliver tactile feedback to proximal areas in stroke survivors who lack sensory integrity in distal areas of their affected arm, but retain motor skills.

## Introduction

The sense of touch is the basis of interaction with other human beings and with the environment around us. The tactile sensation provides information about contact with objects, which is essential to grasp and to manipulate them. Unfortunately, neurological diseases such as stroke can interrupt or damage sensory feedback pathways that normally play a key role in the coordination and accuracy of movements. Depending on stroke severity, from 11 up to 85% of post-stroke patients reported sensory impairments of the upper limb related to the affected area of the brain (Carey et al., 1993; Kim and Choi-Kwon, 1996; Yekutiel, 2000). An impaired somatosensory function has severe negative implications on the quality of daily living. For instance, it can lead to deficits in tactile recognition and fine manipulation of objects as well as to impairments in motor control of the affected limb and problems in adjusting the level of force during grasping (Sullivan and Hedman, 2008; Doyle et al., 2011, 2014; Connell et al., 2014; Hill et al., 2014). As a result, even when the patients have good residual motor functions, the lack of sensory feedback in 40% of the cases leads to a significant decrease in the spontaneous use of the affected limb that contributes to the phenomenon known as *learned non-use* (Dannenbaum and Dykes, 1988; Rand et al., 2001). Consequently, although people suffering from the loss of touch sensation are able to move their limbs, they must rely mainly on visual feedback during daily living activities. Therefore, due to the impaired motor control and the long processing delays of the visual system, even the simplest movements require great concentration and can become nearly impossible (Cameron et al., 2014). Hence, this condition limits the independence of patients, their safety and often prolongs hospital stay (Carey, 1995; Sommerfeld and von Arbin, 2004; Tyson et al., 2008). Therefore, the functional recovery of the upper extremity is one of the primary goals of rehabilitation programs and the additional somatosensory deficits significantly affect the likelihood of achieving higher levels of motor restoration (Patel et al., 2000; Lee et al., 2010). Feedback of tactile information has the potential to improve hand function in patients with sensory loss since it provides additional information that would otherwise be unavailable, thus countering the *learned non-use* phenomenon and favoring functional recovery.

One way to provide feedback of sensory information in patients with sensory loss is through sensory substitution. The substitution can be implemented by exploiting a sensory channel different from the one that is normally used (e.g., substitute vision with touch) or through the same channel but in a different modality (e.g., substitute pressure with vibration or electrotactile stimulation) or involving a different part of the body (e.g., substitute digit with forearm) (Bach-y-Rita and Kercel, 2003). Many approaches have been proposed to elicit tactile sensations, including invasive or non-invasive methods, and they have been widely applied to restore sensory feedback in prosthetic limbs (Antfolk et al., 2013b; Svensson et al., 2017). Providing somatosensory feedback in prosthetics has been shown to improve the utility as well as facilitate the embodiment of the assistive systems (D'Alonzo et al., 2014a; Clemente et al., 2016; Markovic et al., 2018). The feedback can be provided invasively, by interfacing directly the nerves or non-invasively, by applying stimulation to the skin. Prevalent non-invasive techniques are vibrotactile and electrotactile stimulation that deliver mechanical vibration or low-intensity current pulses to the skin in order to provide information about the grasp force (artificial exteroception) and/or joint position (artificial proprioception) (Kaczmarek et al., 1991). For example, the prosthesis grasping force can be communicated by modulating stimulation intensity and/or frequency, i.e., the higher the grasping force, the stronger/faster is the stimulation (Antfolk et al., 2013a). Alternatively, when multiple stimulation channels are available (Štrbac et al., 2016; Dosen et al., 2017), the feedback can be transmitted by changing stimulation location (active channel), which is known as spatial coding. Electrotactile stimulation, as compared to vibrotactile, is particularly suitable for this kind of application since it has low latency, it is energy efficient, it can be delivered in an unobtrusive way and the electrodes are small enough to be worn under clothing. Recent research presented flexible matrix of electrodes to provide spatially distributed electrotactile stimulation to mimic the distributed nature of biological tactile feedback (Štrbac et al., 2016; Franceschi et al., 2017). On the other hand, the large variability in perceived sensation intensity leads to the necessity of a careful and time-consuming calibration.

Although stroke incidence is much higher compared to amputation (e.g., in Italy 200,000 new stroke cases and 3,600 upper limb amputations occur each year), the use of sensory substitution technologies in stroke survivors is far less explored (Kita et al., 2011, 2013; Malešević et al., 2012; Tzorakoleftherakis et al., 2015; Béjot et al., 2016; Imbinto et al., 2016). One of the reasons is the inhomogeneity characterizing the post-stroke status that inevitably complicates the implementation of the substitution feedback. The somatosensory deficits in fact change depending on the size and extent of the injury and clinical cases where the sensory deficits are such as to be disabling for a patient with a good residual limb strength are rather rare (Martin et al., 2002; Hatem et al., 2016). On the other hand, the electrostimulation technique might be even more effective in stroke survivors with a view to plastically reactivate brain areas compromised after the injury. Hence, it becomes essential to investigate which features in the stimulation encode the most relevant output to induce beneficial plasticity.

Furthermore, the amount of information that can be encoded by electrotactile stimulation will depend on the user's ability to discriminate different stimuli. Therefore, exploring the ability to discriminate the number of tactile stimuli delivered over the body surface and the factors that can successfully improve this discrimination is of paramount importance. Several studies have demonstrated that subject's ability to process multiple tactile stimuli delivered over the body surface, or even across the fingertips, is limited. That is, people are simply unable to enumerate accurately more than two or three tactile stimuli applied simultaneously (Gallace et al., 2006, 2008; Riggs et al., 2006; Wang et al., 2018). The human accuracy in tactile enumeration tasks decreased as the number of tactors activated increased. Gallace and coauthors also observed that when the tactile stimuli were presented simultaneously and repeatedly (the stimuli were intermittently turned on and off for a few seconds), the accuracy of the numerosity judgment improved compared to the simple, simultaneous, presentation of the tactile stimuli (Gallace et al., 2006). However, the authors used longer durations in intermittent compared to simultaneous stimulation which makes it difficult to understand the nature of the advantage of the intermittent stimulation. The comparison between simultaneous and sequential presentation of the stimuli was investigated in two recent studies (i.e., Wentink et al., 2011; Boldt et al., 2014). The results showed an advantage of sequential stimulation when estimating the number of active channels. However, the authors applied the stimuli at a different body location than ours (i.e., upper leg or hand). More importantly, they only tested up to three sequential or simultaneous stimuli which is a low upper bound in numerosity judgment tasks (Gallace et al., 2006). Finally, they did not include an intermittent stimulation condition in their protocol.

In the present study, we investigated whether the judgment of numerosity of electrotactile stimuli administered on the shoulder and back is influenced by the tactile code used (simultaneous-intermittent - sequential). By implementing four tactile codes in which total duration, temporal synchrony, and energy of stimulation covaried, we performed a series of enumeration tasks that quantified the subjects' ability to discriminate electrotactile stimuli. To the best of our knowledge, this is the first study in which these three parameters (total duration, synchrony, and energy) have been modulated and compared in a systematic manner. The final goal was to identify an intuitive tactile code which boosts the accuracy of identifying the number of perceived stimuli. Two main hypotheses have been tested in the present study. First, we hypothesized that the tactile numerosity judgment is modulated by the stimulation synchrony. We expected that sequential electrotactile stimulation might lead to better performance. Second, based on the contributions of working memory and attention on perceptual decision-making, we hypothesized that the tactile numerosity judgment would be modulated by the total duration of the electrotactile stimulation (Curtis and D'Esposito, 2003; Wu and Liu, 2008).

Furthermore, in the present study we delivered the feedback on the shoulder and back whereas in prosthetics the feedback is usually administered to the residual limb in order to have a self-contained system (stimulation in the socket). As explained above, the ultimate application of this interface is in stroke patients, in whom the proximal areas are less affected by the sensory deficits. In addition, this arrangement meets several needs: for instance, it provides enough space to distribute the electrodes to achieve anatomically congruent representation of the fingers and palm. Furthermore, the successful integration of wearable systems in daily activities must also meet practical and social issues. The body region selected in the present study is readily accessible, does not obstruct any important function and can be easily hidden under clothing. Finally, the shoulder positioning also enables mimicking some social gestures such as tapping on the shoulder for guiding or alerting. To the best of our knowledge, the perception and discrimination of electrotactile stimuli applied to these areas have been rarely investigated compared to other more distal arm segments. For example, previous studies investigated electrotactile spatial acuity on the shoulder and on the back of the neck (Solomonow et al., 1977; Marcus and Fuglevand, 2009) but without focusing on enumeration task.

In summary, the aim of the present study was to determine how to manipulate temporal electrotactile stimulation parameters, such as Inter-Stimulus Interval (ISI) of sequential stimuli and/or duration of the stimulus application to the skin, to improve the numerosity judgment capability.

## Materials and Methods

### Participants

Ten healthy participants (five males and five females) with no known cognitive or tactile deficits took part in the experiment as volunteers. Participants age ranged from 25 to 31 (mean age: 27 ± 2 years). All participants were naïve to the purpose of the study. The experiments were approved by the Region Liguria Ethical Committee (approval ID 172REG2016, approval date September 13, 2016).

### Electrodes Placement

**Figure 1:**
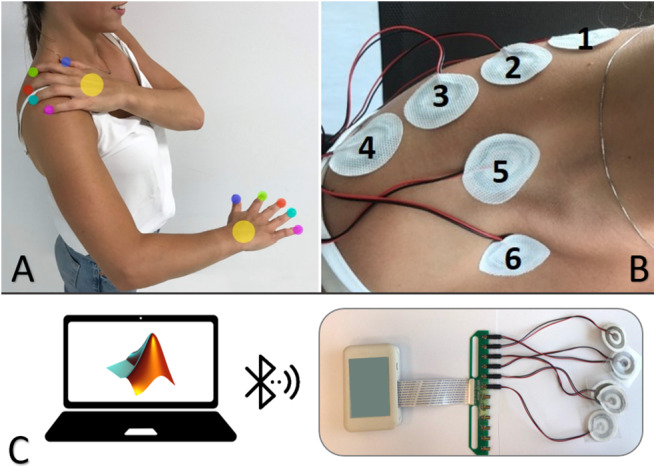
Electrotactile configuration showing the intuitive mapping between the fingers/palm and the electrodes' sites on the shoulder and back **(A)**. Placement of the electrodes on the participants' body **(B)**. Experimental set-up comprising a standard desktop computer (host PC) equipped with a Bluetooth Low Energy (BLE) module and a current-controlled multichannel electrotactile stimulator equipped with sixself-adhesive concentric electrodes **(C)**.

The electrotactile configuration included six electrodes placed on the right (dominant) shoulder and back as shown in Figure 1. In particular, four electrodes were distributed equidistantly (5 cm in between) on the backside of the shoulder along a horizontal line from the base of the neck to the end of the shoulder and two on the front side (one above and one below the collarbone). The inter-pad distance is well above the two-point discrimination threshold for electrical stimulation on the shoulder (Solomonow et al., 1977).

Pulse width, frequency and amplitude were kept constant for the entire duration of the experiment. Specifically, the pulse width was set to 300 μs and the frequency to 100 Hz. This frequency was selected since previous studies demonstrated that it elicited a well-localized, continuous sensation (i.e., responses to individual pulses fused together) resembling constant pressure on the surface of the skin (Wang et al., 2013; D'Alonzo et al., 2014b, 2018; Xu et al., 2015; Štrbac et al., 2016).

### Experimental Setup

The experiment was conducted in a normally illuminated and quiet room. Participants were comfortably seated on a chair in front of a table for the duration of the experiment. The experimental setup (Figure 1C) comprised the following components: (1) current-controlled multichannel electrotactile stimulator prototype WESP (produced by Global Electronics), which incorporates technology for time and space distribution of stimuli introduced by Tecnalia with the IntFES system (Malešević et al., 2012) and previously adapted for tactile feedback applications with MaxSens (Štrbac et al., 2016); (2) a set of six electrodes (CoDe 2.0 C, Spes Medica, Genoa, Italy, http://www.spesmedica.com); and (3) a standard desktop computer (host PC) equipped with a Bluetooth Low Energy (BLE) module for communication with the WESP prototype. The stimulation system generated current-controlled biphasic stimulation pulses with pulse intensity in the range of 0–100 mA (0.1 mA increments), pulse width from 50 to 500 μs and pulse rate between 1 and 400 Hz. The unit integrated 12 stimulation channels with individually and independently adjustable pulse width and amplitude, whereas the pulse rate was a global parameter common to all channels. In addition, the delay between a positive and negative pulse is fixed by the construction of the stimulator, and therefore it cannot be adjusted. The parameters could be set online by sending simple text commands to the stimulator. The stimulator was interfaced via Bluetooth to a portable laptop computer running a custom script within the MATLAB R2018a computing environment (MathWorks Inc., Natick MA). Six self-adhesive disposable electrodes were used to deliver the stimulation. Each electrode consisted of an inner circle and an outer ring arranged in a concentric configuration. The diameter of the inner circle was 10 mm while the outer diameter of the external ring was 30 mm with 5 mm of separation between the two; the thickness was about 1.5 mm (conductive pad: 1 mm, adhesive material: 0.5 mm). During the experiments, the participants were comfortably seated in an adjustable-height chair in front of a table and the stimulator unit was positioned on the arm fixed by an adhesive strip.

### Temporal Encoding Schemes

**Figure 2:**
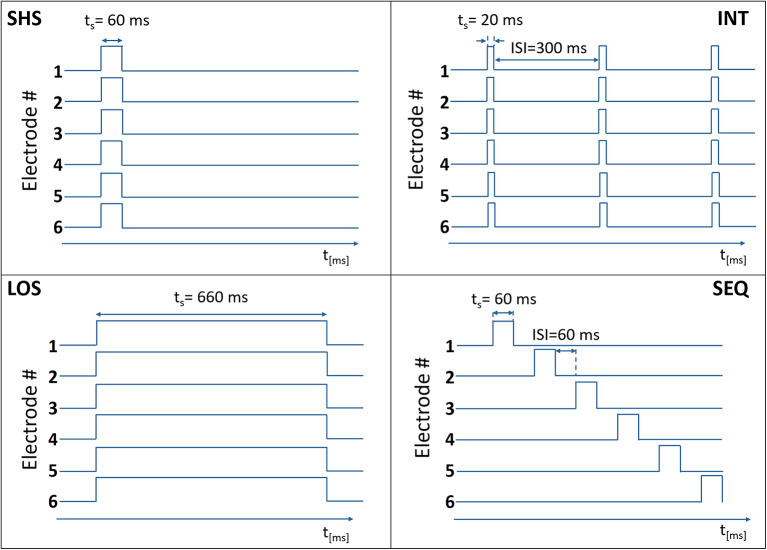
Temporal activation of electrodes in the four electrotactile codes with the time on the x-axis (t_s_ = stimulation time) and the electrode state (0 – non-active, 1 – active) on the y-axis. In this example, six electrodes were activated.

In each trial, a predefined number of electrotactile stimuli (from 1 to 6) was presented to the subject by activating the selected number of electrodes, as described in the protocol (see Experimental Procedure). Four different electrotactile codes (Figure 2) were used to define the timing of electrode activation:

*Short Simultaneous Stimulation (SHS)*. The selected electrodes were activated concurrently for 60 ms.

*Long Simultaneous Stimulation (LOS)*. The selected electrodes were activated concurrently for 660 ms.

*Intermittent Stimulation (INT)*. The selected electrodes were activated concurrently three times for 20 ms with a fixed pause of 300 ms between successive activations.

*Sequential Stimulation (SEQ)*. The selected electrodes were activated sequentially (one after the other) for 60 ms with a variable ISI that depended on the total number of electrodes to be activated (N).

$$\begin{array}{l} ISI\left[ms\right]=\frac{660-N\;*\;60}{N-1} \end{array}$$

**Figure 3:**
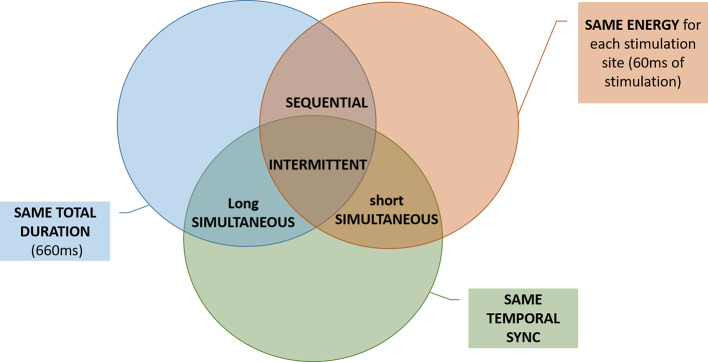
Venn diagram showing differences and similarities (total duration, energy, and temporal synchrony) between the electrotactile codes. For example, the SEQ and the INT code differed only in temporal synchrony, while they have in common the total duration and the energy.

The SEQ, INT, and SHS codes delivered the same amount of energy to each stimulation site. In this context, same energy means that in each trial the subject has received a tactile stimulus at the same level of perceived intensity (as explained in sections Threshold Estimation Phase and Equalization Phase) for the same amount of time (60 ms in SEQ and SHS, 3 × 20 ms in INT). SEQ, INT, and LOS had the same duration per single trial. LOS, INT, and SHS shared the same type of temporal activation (all the electrodes were activated at the same time) (see Figure 3).

### Experimental Procedure

**Figure 4:**
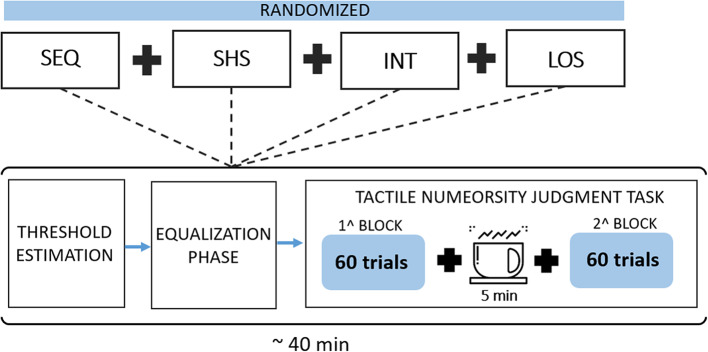
Flow chart of the experimental procedure. Subjects performed a set of four randomized sessions (one for each electrotactile code: SEQ -SHS-INT-LOS). Each session included three phases: a threshold estimation phase, an equalization phase and a tactile numerosity judgment task.

The flow chart of the experimental procedure is shown in Figure 4. Each participant took part in four experimental sessions, one for each electrotactile code (SHS, LOS, INT, and SEQ), separated for at least 1 day and scheduled within 7 days. The order of the electrotactile codes was counter balanced across subjects to minimize training effects. Each session lasted about 40 min and comprised three phases: threshold estimation, equalization and tactile numerosity judgment task.

After positioning the electrodes, the goal of the threshold estimation and equalization phases was to adjust the stimulation intensity for each electrode across electrotactile codes in order to provide a well-perceivable and balanced localized sensation, below the discomfort threshold.

#### Threshold Estimation Phase

First, we estimated the detection threshold (DT) for the electrode 1 (see Figure 1) using a 1-up and 1-down staircase procedure, where the current amplitude is changed trial by trial according to the subject's response. Starting from a subthreshold current amplitude (0.5 mA), we automatically increased the amplitude, with a step-size of 0.1 mA, until the subject reported that he/she felt the stimulus. Each time the stimulus was detected, the current's intensity decreased by the same step-size. Response reversals, i.e., the points at which the subject response changed direction, were recorded. We stopped the procedure when we reached six response reversal, and the detection threshold was determined as the average of the amplitude values corresponding to the last four reversals. Finally, the amplitude for the electrode 1 was set to 3 × DT and kept constant during the experiments. This amplitude was selected based on a pilot study showing that it elicits a clear, comfortable and well-localized sensation. This amplitude was also adopted as the reference stimulus (RS) for electrode 1 (see next section).

#### Equalization Phase

The purpose of this phase was to adjust the stimulation amplitude across the other five electrodes so that the subject perceived similar intensity across all electrodes. To do so, participants performed five 2-intervals forced-choice (2IFC) tasks, one for each electrode from 2 to 6 considering that the amplitude of electrode 1 was determined in the previous phase. In the 2IFC discrimination task, two stimuli - the RS at one electrode (RS*k*, where *k* = 1,…,5 indicates the electrode number) and the test stimulus at a neighboring electrode - were presented one at a time in two successive intervals with an ISI of 1 second, and with the order of presentation varying randomly from trial to trial. The RS number *k* changed as a function of the number *k* of the 2IFC task (*k* from 1 to 5). The corresponding neighboring electrode for each 2IFC task was the electrode *k* + 1. In the first 2IFC, the RS was the electrode number 1 while the neighboring pad was the electrode number 2 (to be determined); in the second 2IFC, the RS was the electrode number 2 (whose amplitude was just been determined) and the neighboring pad was the electrode number 3 (to be determined) and so on. In each trial, participants had to report which interval contained the stronger stimulus. The current amplitude of the RS was kept constant, while the amplitude of the neighboring stimulus varied from trial to trial. The neighboring stimulus was initially set equal to a third of the reference one, and was increased or decreased in steps of 0.1 mA depending on participants' response. As in the threshold estimation phase, we stopped the procedure when six reversals were reached. After that, the experimenter activated the pads in sequence and, whenever necessary, small adjustments in current amplitudes were made.

#### Tactile Numerosity Judgment Phase

After the equalization phase, participants performed a *tactile numerosity judgment task*. In each trial, a random number of electrodes (from 1 to 6) was activated. For each number of electrodes, different activation patterns were chosen randomly among all the possible combinations. For instance, when 2 electrodes were activated, 15 combinations were possible (e.g., 2 and 5 or 1 and 3, and so on). Participants were asked to report how many tactile stimuli they felt (from 1 to 6) in each trial. Response accuracy rather than speediness was stressed. This phase comprised two blocks of 60 trials each with a 5-min break between the blocks. Each number of active electrodes (i.e., 1–6) was presented for 20 trials giving rise to a total of 120 trials. This phase lasted about 20/30 min. In each session, we have tested one of the proposed encoding schemes (SHS, INT, LOS, and SEQ).

### Data Analysis

The three outcome measures were electrotactile intensity threshold, accuracy and deviation. The electrotactile intensity thresholds represent the current amplitudes that were perceived as equal across electrodes and electrotactile codes. The accuracy was defined as the percent success rate in identifying the number of presented stimuli. The deviation was defined as the difference, in terms of the number of electrodes, between the participant's response and the correct answer. The deviation allows identifying potential bias in estimating the number of electrodes (e.g., over/underestimation).

We used the Shapiro-Wilk test to assess the normality of the data distributions. Most outcomes' distributions violated the assumption of normality. Hence, we used non–parametric tests, namely Friedman tests as alternative to the repeated measures ANOVA and, when required, Wilcoxon signed-rank tests for *post-hoc* pairwise comparison (with false discovery rate correction).

Firstly, we measured the electrotactile intensity thresholds for each electrode and then we analyzed the extent to which this variable varies across the shoulder and back based on the electrode position and the electrotactile code. We applied two separate Friedman tests with stimulation code and electrode position as within subjects' factor, respectively.

Mean response accuracy and mean deviation were calculated for each number of active electrodes and each coding scheme. To test our first hypothesis that the subject ability to determine the number of tactile stimuli depends on the number of delivered stimuli, we used Friedman tests applied separately to accuracy and deviation with the number of active electrodes as within subjects' factor. Furthermore, to investigate whether the distance between electrodes might affect the performance, we also compared the accuracy across all the possible electrodes pairs. For instance, we could expect that closer electrodes (e.g., 1 and 2) might lead to a lower accuracy than farther electrodes (e.g., 1 and 4). Similarly, electrodes on the same side of the body (e.g., 1 and 4) might result in lower accuracy than electrodes on the opposite sides of the body (e.g., 1 and 6). Specifically, we ran a Friedman test with all the possible pairs as factor.

To test the second hypothesis that the tactile numerosity judgment is modulated by the coding scheme, we applied Friedman tests to accuracy and deviation with stimulation code as within subjects' factor. Moreover, to evaluate the strength of the obtained results in terms of the magnitude of the difference in the means scores of the groups, we estimated the effect size *r* for each Wilcoxon signed-rank test using the formula *r* = $\frac{z}{\sqrt{n}}$. As for the interpretation of the effect sizes, we followed Cohen (Cohen, 1988). According to his guidelines, small, medium, and large effects correspond to *r* > 0.1, *r* > 0.3, and *r* > 0.5, respectively.

To investigate the interaction between the two factors, number of active electrodes and type of tactile code, we ran a Friedman test for each number of electrodes activated with the electrotactile code as within factor.

Results were also presented in the form of confusion matrices so that we could evaluate the overall performance and identify prevalent mistakes.

Statistical analysis was conducted in Python (Python Software Foundation). The threshold for the statistical significance was set to *p* < 0.05.

## Results

### Current Amplitudes Across Electrodes and Codes

The distribution of electrotactile intensity thresholds was submitted to two Friedman Tests with electrodes location and electrotactile code as factors.

**Figure 5:**
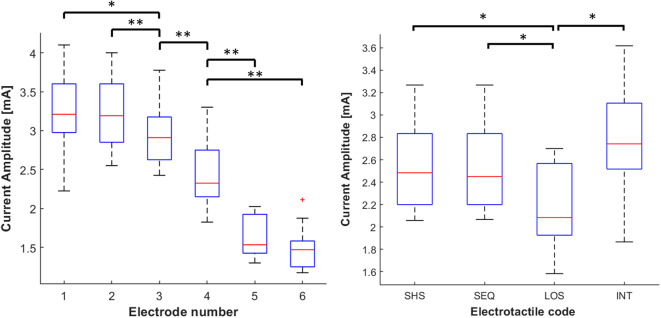
Distribution of current intensities. The data is grouped by the electrodes location (**left** panel) and tactile code (**right** panel). The current intensities is visualized using boxplots, depicting the overall median (horizontal red line), interquartile range (box), maximal/minimal values (whiskers), and outliers (red crosses). The data is grouped by the electrodes location (**left** panel) and tactile code (**right** panel). Asterisks indicate statistical differences. *p < 0.05; **p < 0.01.

The first analysis revealed the main effect of the electrodes location on the current intensity (χ^2^ = 45.8, *p* < 0.001). Particularly, the distribution of current intensities indicated a progressive decrease when moving toward the shoulder. The intensity decreased even more on the frontal side (electrodes 5 and 6) suggesting that this side is significantly more sensitive compared to the back. *Post-hoc* analyses showed that the current intensity at each location differed significantly from all the others (*p* < 0.05, *r* > 0.7), except for the comparison 1–2 and 5–6 (see left panel of Figure 5).

The results of the second Friedman test showed a main effect of the tactile code on the current intensity (χ^2^ = 15.3, *p* < 0.01). *Post-hoc* analyses revealed that the average current amplitude was significantly lower in LOS (2.14 ± 0.18 mA) compared to SHS (2.5 ± 0.21 mA), SEQ (2.5 ± 0.20 mA), and INT (2.76 ± 0.23; *p* < 0.05 and *r* > 0.75 in all cases). This means that lower intensities were required in LOS than in other codes. This result was expected since the effective duration of stimulation was 660 ms, which was much higher than that used in SHS, SEQ (60 ms), and INT (20 ms). No significant differences emerged between the other electrotactile codes (see right panel of Figure 5).

### Numerosity Judgment Across Number of Active Electrodes

**Figure 6:**
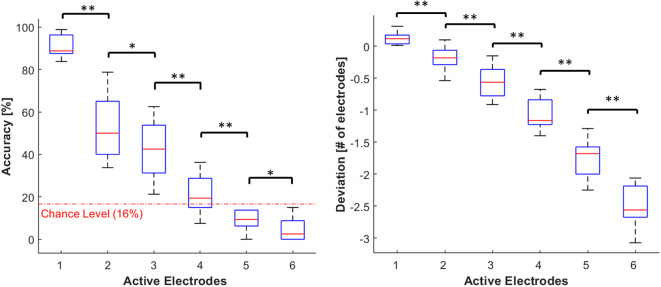
Distribution of accuracy (**left** panel) and deviation (**right** panel). The data is grouped by the number of active electrodes (from 1 to 6). Both the outcome parameters are visualized using boxplots, depicting the overall median (horizontal red line), interquartile range (box), maximal/minimal values (whiskers), and outliers (red crosses). The dotted red line in the accuracy's plot represent the chance level (16%). Asterisks indicate statistical differences. *p < 0.05; **p < 0.01.

Accuracy data were submitted to a Friedman Test with the numerosity (six levels: from 1 to 6) as a factor and the analyses revealed a significant effect (χ^2^ = 48.21 *p* < 0.001). Particularly, accuracy decreased as the number of active electrodes increased. *Post-hoc* analyses showed that the accuracy at each level of numerosity differed significantly from all the others (*p* < 0.05 and *r* > 0.7 in all cases) (see Figure 6). When the number of active electrodes was 5 or 6, participants' responses were compatible with a chance performance (accuracy around 16%).

Similarly, the deviation measures were submitted to a Friedman Test with the numerosity as a factor, and we found a significant main effect (χ^2^ = 50, *p* < 0.001). The underestimation increased with the number of active electrodes. *Post-hoc* analyses showed that the deviation at each level of numerosity differed significantly from all the others (*p* < 0.01 and *r* > 0.9 in all cases) (see Figure 6).

In addition, accuracy data were submitted to a Friedman test with all the possible pairs as factor and the analysis revealed that performance was uniform across all possible electrodes pairs (χ^2^ = 14.1 *p* = 0.44). This finding showed that the distance between electrodes was appropriate and that the configuration did not favor the recognition of a specific electrode pair.

### Numerosity Judgment Across Electrotactile Codes

**Figure 7:**
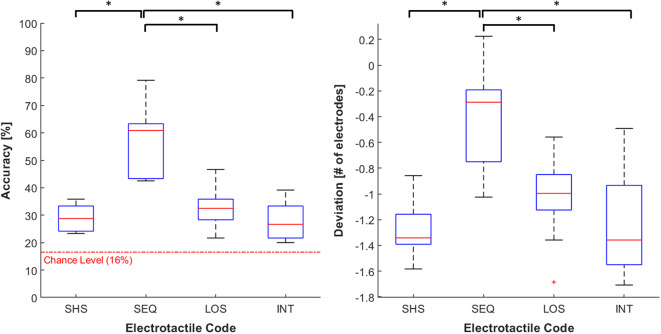
Distribution of accuracy (**left** panel) and deviation (**right** panel). The data is grouped by the four feedback codes (SHS, SEQ, LOS, and INT). Both the outcome parameters are visualized using boxplots, depicting the overall median (horizontal red line), interquartile range (box), maximal/minimal values (whiskers), and outliers (red crosses). The dotted red line in the accuracy's plot represent the chance level (16%). Asterisks indicate statistical differences. *p < 0.05.

The Friedman test showed the main effect of the electrotactile code on the accuracy (χ^2^ = 22.72 *p* < 0.001). *Post-hoc* analyses revealed that the average accuracy was significantly higher in SEQ (57 ± 12%) compared to SHS (29 ± 5%), LOS (33 ± 7%), and INT (28 ± 7%; *p* < 0.05 and *r* > 0.97 in all cases). No significant differences emerged between the other electrotactile codes. However, we observed a trend toward higher accuracy in LOS compared to SHS (*p* = 0.063; see Figure 7).

For the deviation, we observed a similar trend. Specifically, the electrotactile code significantly affected the deviation (χ^2^ = 21.36 *p* < 0.001). The average underestimation in SEQ (−0.389 ± 0.37) was significantly closer to zero compared to SHS (−1.28 ± 0.22), LOS (−1.03 ± 0.32) and INT (−1.25 ± 0.39; *p* < 0.05 and *r* > 0.91 in all cases). No significant differences emerged between the other electrotactile codes. However, the underestimation tended to be lower in LOS than in SHS (*p* = 0.055; see Figure 7).

### Interaction Between Number of Electrodes and Electrotactile Codes

**Figure 8:**
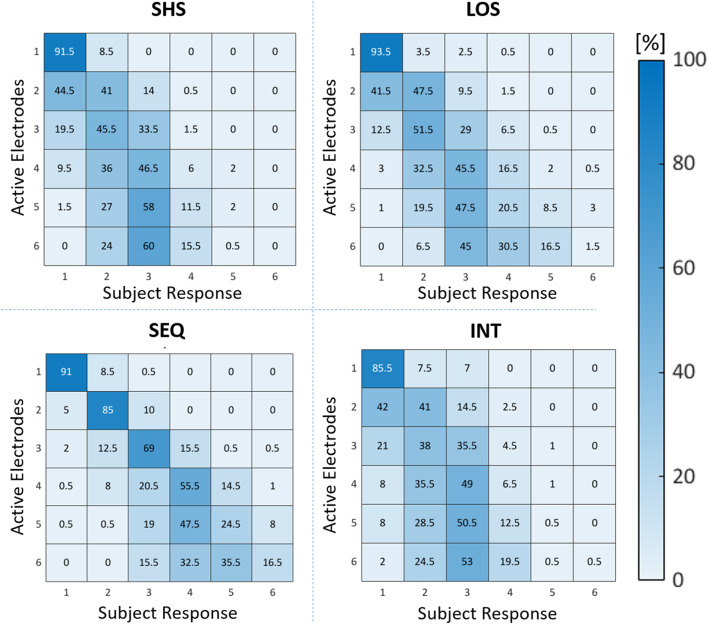
Confusion matrices for recognition of number of active electrodes for each electrotactile code (SHS, SEQ, LOS, and INT), the number inside the cells represent the sum of the ten subjects' results. The entries in the main diagonal cells represent the number of trials in which participants correctly enumerated the number of active electrodes. The entries off the main diagonal represent instead trials in which a wrong response was done. The cells in the triangle above the main diagonal represent the number of trials in which the subjects overestimated the number of active electrodes, while the cells in the triangle below the main diagonal represent the number of trials in which participants underestimated the number of active electrodes. The darker the blue color, the more likely it is the answer.

The four confusion matrices reported in Figure 8 describe the distribution of mistakes for each electrotactile code. A closer examination of the SEQ confusion matrix reveals that the entries just next to the main diagonal cells are generally the highest compared to those in the cells further from the main diagonal, suggesting a gradual accuracy degradation. This means that the participants were more inclined to misjudge the number of active electrodes by one at most. In other words, when the subjects were wrong the answers were not given randomly but they were generally close to the correct answer. This trend is less evident in the other three electrotactile codes, where participants reported they felt 2 or 3 stimuli even if the number of stimuli presented exceeded three. By comparing the sum of all entries in the triangle above the main diagonal and the one below the main diagonal, we confirmed that, in general, participants made more underestimation than overestimation mistakes. Furthermore, the correct answer was the most likely when 1–4 electrodes were activated sequentially. Instead, an underestimation of 1 electrode is most likely to happen when 5 and 6 electrodes are sequentially activated.

Results on the interaction between electrotactile code and electrodes number showed higher accuracy when using SEQ compared to SHS, LOS, and INT whenever 2–5 electrodes were activated. Similarly, the deviation in SEQ was lower compared to SHS, LOS, and INT whenever 3–6 electrodes were activated. Furthermore, we observed a significant difference between LOS, INT, and SHS when six electrodes were activated (described in detail in the Supplementary Materials).

## Discussion

This is the first study comparing three different tactile codes, i.e., simultaneous, intermittent, and sequential in a numerosity judgment task. The main finding of our study is that the sequential stimulation elicited a significantly higher accuracy in judging the number of activated electrodes compared to the simultaneous and intermittent condition. The general trend in all conditions was toward an underestimation of the number of activated channels, i.e., the perceived number of active channels was mostly three when more than three channels were actually active in the simultaneous and intermittent conditions, confirming the results of previous studies (Gallace et al., 2006; Riggs et al., 2006; Wang et al., 2018). However, this underestimation was significantly lower in the sequential condition. In fact, when the channels were sequentially activated the participants were able to perceive up to four stimuli with a good level of accuracy and they also made less mistakes when perceiving five and six stimuli. Importantly, the underestimation in our task cannot be due to a sensory funneling effect since the channels in our setup were farther apart than 2–3 cm (Von Spsycho-Acoustic, 1959). Therefore, our first hypothesis about the modulation effect of the stimulus synchronicity confirmed to be correct.

This result extends what is already known about the comparison between simultaneous and sequential stimulation in tactile discrimination tasks (Wentink et al., 2011; Boldt et al., 2014). Wentink and co-authors applied up to three simultaneous or sequential vibrotactile stimuli on the leg and asked participants to estimate number and location of stimuli. They found an advantage for the sequential condition. However, they used a different type of stimulus (i.e., vibration), a different body location and they imposed a lower maximum number of activated stimuli (i.e., 3). The last point seems to be a major limitation in numerosity judgment tasks because the previous studies have shown good estimation ability when up to three simultaneous stimuli were delivered (e.g., Gallace et al., 2006). Furthermore, there are also evidences that subjects can subitize up to three tactile stimuli administered on the hand (Riggs et al., 2006) suggesting that the numerosity judgment task starts to be more challenging when four or more stimuli are delivered. Notably, a similar behavior has been observed in the visual modality in which increasing the number of items above four produced larger response latencies and error rates (Atkinson et al., 1976). The fact that the sequential stimulation led to a better numerosity discrimination is consistent with the high temporal discrimination of tactile perception which might prefer serial information (Bach-y-Rita et al., 1969; Lechelt, 1975; Gallace et al., 2006), at least when compared to vision (Lechelt, 1975), also because few locations can be simultaneously processed by touch without strongly affecting the performance (Craig, 1985). Several theories have been suggested to explain this effect. Since the underestimation is evident also when stimulating very far body locations it might not be due to cutaneous masking (von Békésy, 1959; Alluisi et al., 1965) but to other phenomena such as central masking, limitations of spatial attention, or short-term memory (e.g., Miller, 1956; Alluisi et al., 1965; Fisher, 1984; Cowan, 2001; Hillstrom et al., 2002). In support of this hypothesis, we found no significant differences in performance considering different electrode pairs. In fact, if the cutaneous masking explanation was true, we would have expected a lower numerosity judgment accuracy when closer and/or same-side electrodes were activated. This was not the case and electrodes separated up to 18 cm were not discriminated better than electrodes separated by only 5 cm. Additionally, we remind that the minimum distance we used between channels was well above the two point discrimination threshold (Solomonow et al., 1977). Therefore, based on these data, as well as the observation from previous studies (e.g., Gallace et al., 2006), the underestimation in numerosity judgment seems to be due to an higher level phenomenon such as central masking or limitation of spatial attention. Hence, the accuracy improvement we observed in the sequential code might be due to the enhanced capacity in shifting the attention toward the sequentially activated spatial locations.

Interestingly, the two continuous and intermittent codes did not differ significantly. This result seems to be in contrast with a previous finding showing an advantage of the intermittent over the simultaneous stimulation (Gallace et al., 2006). However, Gallace et al. in their study compared a single burst of 200 ms to an intermittent stimulation composed of several 200 ms bursts in a 5 s time window. Therefore, their effect might be due to the perceptual facilitation in discriminating the number of tactile stimuli when judging a much longer temporal sequence of stimulation rather than to an intrinsic difference between tactile code used (e.g., simultaneous vs. intermittent). However, as compared to Gallace et al. we could not find an effect of stimulus duration in our numerosity judgment task. For instance, the performance in discriminating the number of active stimuli was not different in the short and long simultaneous codes. This might be simply due to the different temporal range in our studies. Our longest sequence is indeed much shorter than the longest sequence in Gallace's study (0.66 vs. 5 s). By contrast, in our study the stimulus duration significantly affected the electrotactile intensity thresholds, in fact, a lower current amplitude was necessary to provide the same tactile sensation when using the long simultaneous compared to the other codes. We have selected the duration in the present study considering the envisioned future applications of electrotactile feedback in sensory substitution. In this case, it is of interest to transmit a tactile message with a short delay so that the subject can react to the perceived feedback information with an appropriate control action. This seems to suggest that part of the mistakes in judgment might be due to the difficulty in counting the number of stimuli when brief sequences of stimulation are delivered. Therefore, our second hypothesis about the effect of total duration revealed to be false.

Another result of our study is that the energy per stimulation does not have an effect in numerosity judgment. In fact, we did not find a difference between long simultaneous and intermittent code which shared the same total duration and temporal synchrony but only differed in the energy per stimulation site. Similarly, the difference between sequential and intermittent codes which shared the same total duration and energy reinforces the idea that the relevant dimension explaining the effect is the synchrony/asynchrony of the stimulation.

Furthermore, the distribution of accuracy and deviation graph by varying the number of active electrodes showed the strong homogeneity among the subjects (highlighted by a very low variance). This result might allow us to predict the performance of a healthy subject during a numerosity judgment task depending on the number of active electrodes and, consequently, to define a baseline for the clinical campaign. There are several potential limitations in this study. One limitation might arise from the limited size of our sample. Nevertheless, to overcome this issue, further analysis relative to the effect size of the results were carried out. The main effects we found were ranging between “large” and “very large,” suggesting a high reliability of these findings. Thus, we believe that our results should not be strongly affected by the small sample size. Another limitation might arise from our choice to include only healthy and young participants in this study. The narrow age range of the participants was chosen to obtain results comparable with the previous literature. However, aging has been already proved to be a key factor in the perception of tactile stimuli (Cholewiak and Collins, 2003; Wickremaratchi and Llewelyn, 2006; Lin et al., 2015) and, for this reason, when repeating the same experiment in older people the results could be different. The performance could change even more in post-stroke patients considering their neurological and somatosensory deficits. Hence, future studies might want to validate our findings with stroke survivors. In this sense, the initial investigation in the present work was meant to define the necessary baseline to compare future results and to pave the way for more practical and clinically oriented experiments. Another limitation is the absence of training. An appropriate training may lead to better performance for a higher number of electrodes (Cohen et al., 2018) and reduce the gap of sequential vs. simultaneous codes. Furthermore, such training could be very useful in the clinical campaign with post-stroke patients because it could greatly improve the effectiveness of the re-mapping of the hand over the shoulder. Other limitations might arise from the fact that during the experiments the subjects did not know that it was a re-mapping of their hand over the shoulder and therefore we actually had not activated a real sensory substitution process. Therefore, in order to obtain more indicative results on the practical use of our approach, the association of the single electrode with a single finger should be specified during the experiments. Moreover, it could be interesting to investigate the intuitiveness of this sensory substitution interface analyzing the reaction time and ability in localizing the electrodes. These results will allow us to identify the locations of electrodes associated with a lower localization performance and will allow a subsequent adjustment of the position of the electrodes.

The ultimate objective of this research was to find out the best way to provide touch information using the electrotactile stimulation in order to facilitate the closed-loop control of goal-directed tasks in post-stroke patients. This feedback interface - encoding the hand shape - would be able to deliver information relative to the number of fingers involved in a grasping or pinching task. In particular, we investigated the ability of subjects to interpret the number of tactile stimuli delivered. The results allowed us to identify a tactile code, i.e., sequential stimulation, which could be used as a sensory substitution replacement of the hand over the shoulder in post-stroke patients. Each electrode can indeed represent a finger of a hand plus one more electrode for the palm. As demonstrated in the present study, this stimulation paradigm facilitates the subjects' ability to identify the number of active electrodes, which can improve the effectiveness of sensory substitution feedback.

## Data Availability Statement

The datasets analyzed for this study can be found in the Figshare repository, https://figshare.com/s/158741db19ad7bbd5f16.

## Ethics Statement

The studies involving human participants were reviewed and approved by Region Liguria Ethical Committee (approval ID 172REG2016, approval date September 13, 2016). The participants provided their written informed consent to participate in this study.

## Author Contributions

Testing and data collection were performed by SN. SN performed the data analysis and interpretation under the supervision of FL and LB. SN, FL, and LB wrote the manuscript. SD, LS, MV, and CT reviewed the manuscript. LS and MV wrote the ethical committee proposal. All authors approved the final version of the manuscript for submission, developed the study concept and contributed to the study design.

## Conflict of Interest

LB was employed by company Acoesis Inc. The remaining authors declare that the research was conducted in the absence of any commercial or financial relationships that could be construed as a potential conflict of interest.

## References

[B1] Alluisi E. A. Morgan B. B. Hawkes G. R. (1965). Masking of cutaneous sensations in multiple stimulus presentations. Percept. Mot. Skills 20, 39–45. 10.2466/pms.1965.20.1.39 14286552

[B2] Antfolk C. D'Alonzo M. Controzzi M. Lundborg G. Rosen B. Sebelius F.. (2013a). Artificial redirection of sensation from prosthetic fingers to the phantom hand map on transradial amputees: vibrotactile versus mechanotactile sensory feedback. IEEE Trans. Neural Syst. Rehabil. Eng. 21, 112–120. 10.1109/TNSRE.2012.2217989 23033439

[B3] Antfolk C. D'Alonzo M. Rosén B. Lundborg G. Sebelius F. Cipriani C. (2013b). Sensory feedback in upper limb prosthetics. Expert Rev. Med. Devices 10, 45–54. 10.1586/erd.12.68 23278223

[B4] Atkinson J. Campbell F. W. Francis M. R. (1976). The magic number 4 ± 0: a new look at visual numerosity judgements. Perception 5, 327–334. 10.1068/p050327 980674

[B5] Bach-y-Rita P. Kercel S. W. (2003). Sensory substitution and the human-machine interface. Trends Cogn. Sci. 7, 541–546. 10.1016/j.tics.2003.10.013 14643370

[B6] Bach-y-Rita P. Collins C. C. Saunders F. A. White B. Scadden L. (1969). Vision substitution by tactile image projection. Nature 221, 963–964. 10.1038/221963a0 5818337

[B7] Béjot Y. Bailly H. Durier J. Giroud M. (2016). Epidemiology of stroke in Europe and trends for the 21st century. Press. Med. 45, e391–e398. 10.1016/j.lpm.2016.10.003 27816343

[B8] Boldt R. Gogulski J. Gúzman-Lopéz J. Carlson S. Pertovaara A. (2014). Two-point tactile discrimination ability is influenced by temporal features of stimulation. Exp. Brain Res. 232, 2179–2185. 10.1007/s00221-014-3908-y 24668131

[B9] Cameron B. D. de la Malla C. López-Moliner J. (2014). The role of differential delays in integrating transient visual and proprioceptive information. Front. Psychol. 5:50. 10.3389/fpsyg.2014.00050 24550870 PMC3910305

[B10] Carey L. M. (1995). Review on somatosensory loss after stroke. Crit. Rev. Phys. Rehabil. Med. 29, 1–41. 10.1615/CritRevPhysRehabilMed.v29.i1-4.10

[B11] Carey L. M. Matyas T. A. Oke L. E. (1993). Sensory loss in stroke patients: Effective training of tactile and proprioceptive discrimination. Arch. Phys. Med. Rehabil. 74, 602–611. 10.1016/0003-9993(93)90158-7 8503750

[B12] Cholewiak R. W. Collins A. A. (2003). Vibrotactile localization on the arm: effects of place, space, and age. Percept. Psychophys. 65, 1058–1077. 10.3758/BF03194834 14674633

[B13] Clemente F. D'Alonzo M. Controzzi M. Edin B. B. Cipriani C. (2016). Non-invasive, temporally discrete feedback of object contact and release improves grasp control of closed-loop myoelectric transradial prostheses. IEEE Trans. Neural Syst. Rehabil. Eng. 24, 1314–1322. 10.1109/TNSRE.2015.2500586 26584497

[B14] Cohen J. (1988). Statistical Power Analysis for the Behavioural Science, 2nd Edn. London: Routledge.

[B15] Cohen Z. Z. Aisenberg D. Henik A. (2018). The effects of training on tactile enumeration. Psychol. Res. 82, 468–487. 10.1007/s00426-016-0835-5 28025676

[B16] Connell L. A. McMahon N. E. Adams N. (2014). Stroke survivors' experiences of somatosensory impairment after stroke: an interpretative phenomenological analysis. Physiother 100, 150–155. 10.1016/j.physio.2013.09.003 24239191

[B17] Cowan N. (2001). The magical number 4 in short-term memory: a reconsideration of mental storage capacity. Behav. Brain Sci. 24, 87–114. 10.1017/S0140525X01003922 11515286

[B18] Craig J. C. (1985). Attending to two fingers: two hands are better than one. Percept. Psychophys. 38, 496–511. 10.3758/BF03207059 3834395

[B19] Curtis C. E. D'Esposito M. (2003). Persistent activity in the prefrontal cortex during working memory. Trends Cogn. Sci. 7, 415–423. 10.1016/S1364-6613(03)00197-9 12963473

[B20] D'Alonzo M. Clemente F. Cipriani C. (2014a). Vibrotactile stimulation promotes embodiment of an alien hand in amputees with phantom sensations. IEEE Trans. Neural Syst. Rehabil. Eng. 23, 450–457. 10.1109/TNSRE.2014.2337952 25051556

[B21] D'Alonzo M. Dosen S. Cipriani C. Farina D. (2014b). HyVE: hybrid vibro-electrotactile stimulation for sensory feedback and substitution in rehabilitation. IEEE Trans. Neural Syst. Rehabil. Eng. 22, 290–301. 10.1109/TNSRE.2013.2266482 23782817

[B22] D'Alonzo M. Engels L. F. Controzzi M. Cipriani C. (2018). Electro-cutaneous stimulation on the palm elicits referred sensations on intact but not on amputated digits. J. Neural Eng. 15:016003. 10.1088/1741-2552/aa81e2 28741593

[B23] Dannenbaum R. M. Dykes R. W. (1988). Sensory loss in the hand after sensory stroke: therapeutic rationale. Arch. Phys. Med. Rehabil. 69, 833–839. 10.3233/RNN-150606 3178450

[B24] Dosen S. Markovic M. Strbac M. Belic M. Kojic V. Bijelic G.. (2017). Multichannel electrotactile feedback with spatial and mixed coding for closed-loop control of grasping force in hand prostheses. IEEE Trans. Neural Syst. Rehabil. Eng. 25, 183–195. 10.1109/TNSRE.2016.2550864 27071179

[B25] Doyle S. Bennett S. Fasoli S. McKenna K. (2011). Interventions for sensory impairment in the upper limb after stroke. Stroke 42:e18. 10.1161/STROKEAHA.110.604348 20556766 PMC6464855

[B26] Doyle S. D. Bennett S. Dudgeon B. (2014). Upper limb post-stroke sensory impairments: the survivor's experience. Disabil. Rehabil. 36, 993–1000. 10.3109/09638288.2013.825649 23971679

[B27] Fisher D. L. (1984). Central capacity limits in consistent mapping, visual search tasks: four channels or more? Cogn. Psychol. 16, 449–484. 10.1016/0010-0285(84)90017-3 6509895

[B28] Franceschi M. Seminara L. Dosen S. Strbac M. Valle M. Farina D. (2017). A system for electrotactile feedback using electronic skin and flexible matrix electrodes: experimental evaluation. IEEE Trans. Haptics 10, 162–172. 10.1109/TOH.2016.2618377 27775538

[B29] Gallace A. Tan H. Z. Spence C. (2006). Numerosity judgments for tactile stimuli distributed over the body surface. Perception 35, 247–266. 10.1068/p5380 16583769

[B30] Gallace A. Tan H. Z. Spence C. (2008). Can tactile stimuli be subitised? An unresolved controversy within the literature on numerosity judgments. Perception 37, 782–800. 10.1068/p5767 18605150

[B31] Hatem S. M. Saussez G. della Faille M. Prist V. Zhang X. Dispa D.. (2016). Rehabilitation of motor function after stroke: a multiple systematic review focused on techniques to stimulate upper extremity recovery. Front. Hum. Neurosci. 10:442. 10.3389/fnhum.2016.00442 27679565 PMC5020059

[B32] Hill V. Fisher T. Schmid A. Crabtree J. Page S. (2014). Relationship between touch sensation of the affected hand and performance of valued activities in individuals with chronic stroke. Top. Stroke Rehabil. 21, 339–346. 10.1310/tsr2104-339 25150666

[B33] Hillstrom A. P. Shapiro K. L. Spence C. (2002). Attentional limitations in processing sequentially presented vibrotactile targets. Percept. Psychophys. 64, 1068–1082. 10.3758/BF03194757 12489662

[B34] Imbinto I. Peccia C. Controzzi M. Cutti A. G. Davalli A. Sacchetti R.. (2016). Treatment of the partial hand amputation: an engineering perspective. IEEE Rev. Biomed. Eng. 9, 32–48. 10.1109/RBME.2016.2523799 26849872

[B35] Kaczmarek K. A. Webster J. G. Bach-y-Rita P. Tompkins W. J. (1991). Electrotactile and vibrotactile displays for sensory substitution systems. IEEE Trans. Biomed. Eng. 38, 1–16. 10.1109/10.68204 2026426

[B36] Kim J. S. Choi-Kwon S. (1996). discriminative sensory dysfunction after unilateral stroke. Stroke 27, 677–682. 10.1161/01.STR.27.4.677 8614929

[B37] Kita K. Otaka Y. Takeda K. Sakata S. Ushiba J. Kondo K.. (2013). A pilot study of sensory feedback by transcutaneous electrical nerve stimulation to improve manipulation deficit caused by severe sensory loss after stroke. J. Neuroeng. Rehabil. 10:55. 10.1186/1743-0003-10-55 23764012 PMC3701472

[B38] Kita K. Takeda K. Osu R. Sakata S. Otaka Y. Ushiba J. (2011). A sensory feedback system utilizing cutaneous electrical stimulation for stroke patients with sensory loss. IEEE Int. Conf. Rehabil. Robot. 2011:5975489. 10.1109/ICORR.2011.5975489 22275686

[B39] Lechelt E. C. (1975). Temporal numerosity discrimination: intermodal comparisons revisited. Br. J. Psychol. 66, 101–108. 10.1111/j.2044-8295.1975.tb01444.x 1131477

[B40] Lee S. Y. Lim J. Y. Kang E. K. Han M. K. Bae H. J. Paik N. J. (2010). Prediction of good functional recovery after stroke based on combined motor and somatosensory evoked potential findings. J. Rehabil. Med. 42, 16–20. 10.2340/16501977-0475 20111839

[B41] Lin C. C. Whitney S. L. Loughlin P. J. Furman J. M. Redfern M. S. Sienko K. H.. (2015). The effect of age on postural and cognitive task performance while using vibrotactile feedback. J. Neurophysiol. 113, 2127–2136. 10.1152/jn.00083.2014 25589585 PMC4416543

[B42] Malešević N. M. Maneski L. Z. P. Ilić V. Jorgovanović N. Bijelić G. Keller T.. (2012). A multi-pad electrode based functional electrical stimulation system for restoration of grasp. J. Neuroeng. Rehabil. 9, 1–12. 10.1186/1743-0003-9-66 23009589 PMC3547757

[B43] Marcus P. L. Fuglevand A. J. (2009). Perception of electrical and mechanical stimulation of the skin: implications for electrotactile feedback. J. Neural Eng. 6:066008. 10.1088/1741-2560/6/6/066008 19918109 PMC7738194

[B44] Markovic M. Schweisfurth M. A. Engels L. F. Bentz T. Wüstefeld D. Farina D.. (2018). The clinical relevance of advanced artificial feedback in the control of a multi-functional myoelectric prosthesis. J. Neuroeng. Rehabil. 15, 1–15. 10.1186/s12984-018-0371-1 29580245 PMC5870217

[B45] Martin C. Dellatolas G. Viguier D. Willadino-Braga L. Deloche G. (2002). Subjective experience after stroke. Appl. Neuropsychol. 9, 148–158. 10.1207/S15324826AN0903_3 12584080

[B46] Miller G. A. (1956). The magical number seven plus or minus two: some limits on our capacity for processing information. Psychol. Rev. 63, 81–97. 10.1037/0033-295x.101.2.343 13310704

[B47] Patel A. T. Duncan P. W. Lai S. M. Studenski S. (2000). The relation between impairments and functional outcomes poststroke. Arch. Phys. Med. Rehabil. 81, 1357–1363. 10.1053/apmr.2000.9397 11030501

[B48] Rand D. Gottlieb D. Weiss P. L. (2001). Recovery of patients with a combined motor and proprioception deficit during the first six weeks of post stroke rehabilitation. Phys. Occup. Ther. Geriatr. 18, 69–87. 10.1080/J148v18n03_05

[B49] Riggs K. J. Ferrand L. Lancelin D. Fryziel L. Dumur G. Simpson A. (2006). Subitizing in tactile perception. Psychol. Sci. 17, 271–272. 10.1111/j.1467-9280.2006.01696.x 16623680

[B50] Solomonow M. Lyman J. Freedy A. (1977). Electrotactile two-point discrimination as a function of frequency, body site, laterality, and stimulation codes. Ann. Biomed. Eng. 5, 47–60. 10.1007/BF02409338 851263

[B51] Sommerfeld D. K. von Arbin M. H. (2004). The impact of somatosensory function on activity performance and length of hospital stay in geriatric patients with stroke. Clin. Rehabil. 18, 149–155. 10.1191/0269215504cr710oa 15053123

[B52] Štrbac M. Belić M. Isaković M. Kojić V. Bijelić G. Popović I.. (2016). Integrated and flexible multichannel interface for electrotactile stimulation. J. Neural Eng. 13:046014. 10.1088/1741-2560/13/4/046014 27296902

[B53] Sullivan J. E. Hedman L. D. (2008). Sensory dysfunction following stroke: incidence, significance, examination, and intervention. Top. Stroke Rehabil. 15, 200–217. 10.1310/tsr1503-200 18647725

[B54] Svensson P. Wijk U. Björkman A. Antfolk C. (2017). A review of invasive and non-invasive sensory feedback in upper limb prostheses. Expert Rev. Med. Devices 14, 439–447. 10.1080/17434440.2017.1332989 28532184

[B55] Tyson S. F. Hanley M. Chillala J. Selley A. B. Tallis R. C. (2008). Sensory loss in hospital-admitted people with stroke: characteristics, associated factors, and relationship with function. Neurorehabil. Neural Repair 22, 166–172. 10.1177/1545968307305523 17687023

[B56] Tzorakoleftherakis E. Bengtson M. C. Mussa-Ivaldi F. A. Scheidt R. A. Murphey T. D. (2015). Tactile proprioceptive input in robotic rehabilitation after stroke. Proc. - IEEE Int. Conf. Robot. Autom. 2015, 6475–6481. 10.1109/ICRA.2015.7140109

[B57] von Békésy G. (1959). Similarities between hearing and skin sensations. Psychol. Rev. 66, 1–22. 10.1037/h0046967 13645847

[B58] Von Spsycho-Acoustic G. (1959). Neural Funneling along the Skin and between the Inner and outer hair cells of the cochlea. J. Acoust. Soc. Am. 31, 1236–1249. 10.1121/1.1907851

[B59] Wang D. Peng C. Afzal N. Li W. Wu D. Zhang Y. (2018). Localization performance of multiple vibrotactile cues on both arms. IEEE Trans. Haptics 11, 97–106. 10.1109/TOH.2017.2742507 28841557

[B60] Wang T. Li S. Chai G. Lan N. (2013). “Perceptual attributes of cutaneous electrical stimulation to provide sensory information for prosthetic limb,” in 2013 IEEE 3rd International Conference on Information Science and Technology, ICIST (Yangzhou) 2013, 22–25. 10.1109/ICIST.2013.6747492

[B61] Wentink E. C. Mulder A. Rietman J. S. Veltink P. H. (2011). “Vibrotactile stimulation of the upper leg: effects of location, stimulation method and habituation,” in Proceedings of the Annual International Conference of the IEEE Engineering in Medicine and Biology Society, EMBS (IEEE) (Boston, MA), 1668–1671. 10.1109/IEMBS.2011.6090480 22254645

[B62] Wickremaratchi M. M. Llewelyn J. G. (2006). Effects of ageing on touch. Postgrad. Med. J. 82, 301–304. 10.1136/pgmj.2005.039651 16679466 PMC2563781

[B63] Wu C. Liu Y. (2008). Queuing network modeling of the Psychological Refractory Period (PRP). Psychol. Rev. 115, 913–954. 10.1037/a0013123 18954209

[B64] Xu H. Zhang D. Member S. Huegel J. C. Member S. Xu W. (2015). Effects of different tactile feedback on myoelectric closed-loop control for grasping based on electrotactile stimulation. IEEE Trans. Neural. Syst. Rehabil. Eng. 24, 827–836. 10.1109/TNSRE.2015.2478153 26372430

[B65] Yekutiel M. (2000). Sensory Re-Education of the Hand after Stroke. London: Whurr Publishers.

