# Supplementary material for: Temporal Asynchrony but Not Total Energy Nor Duration Improves the Judgment of Numerosity in Electrotactile Stimulation
Source: Front Bioeng Biotechnol. 2020 Jun 19;8:555. doi: 10.3389/fbioe.2020.00555 (PMC7325877; doi:10.3389/fbioe.2020.00555)
Supplement: Supplementary file 1 [file Data_Sheet_1.docx]

Supplementary Material

# Supplementary Figures and Tables

To investigate the interaction between the two factors, number of active electrodes and kind of tactile code, we ran a Friedman test for each number of electrodes activated with the electrotactile code as within factor (see Tables).

Results on the interaction between electrotactile code and electrodes number showed higher accuracy when using SEQ compared to SHS, LOS and INT whenever 2 to 5 electrodes were activated. Similarly, the deviation in SEQ was lower compared to SHS, LOS, and INT whenever 3 to 6 electrodes were activated. Furthermore, we observed a significant difference between LOS and INT and SHS when six electrodes were activated.

| **Accuracy** |  | Chi-square | p-value |
| --- | --- | --- | --- |
| Number of active electrodes | 1 | 2.36 | 0.5 |
|  | 2 | 15.3 | <0.01** |
|  | 3 | 16.9 | <0.001*** |
|  | 4 | 17.9 | <0.001*** |
|  | 5 | 14.7 | <0.01** |
|  | 6 | 9.43 | <0.05* |

| **Deviation** |  | Chi-square | p-value |
| --- | --- | --- | --- |
| Number of active electrodes | 1 | 2.61 | 0.46 |
|  | 2 | 7.01 | 0.07 |
|  | 3 | 18.9 | <0.001*** |
|  | 4 | 19.32 | <0.001*** |
|  | 5 | 21.36 | <0.001*** |
|  | 6 | 21 | <0.001*** |


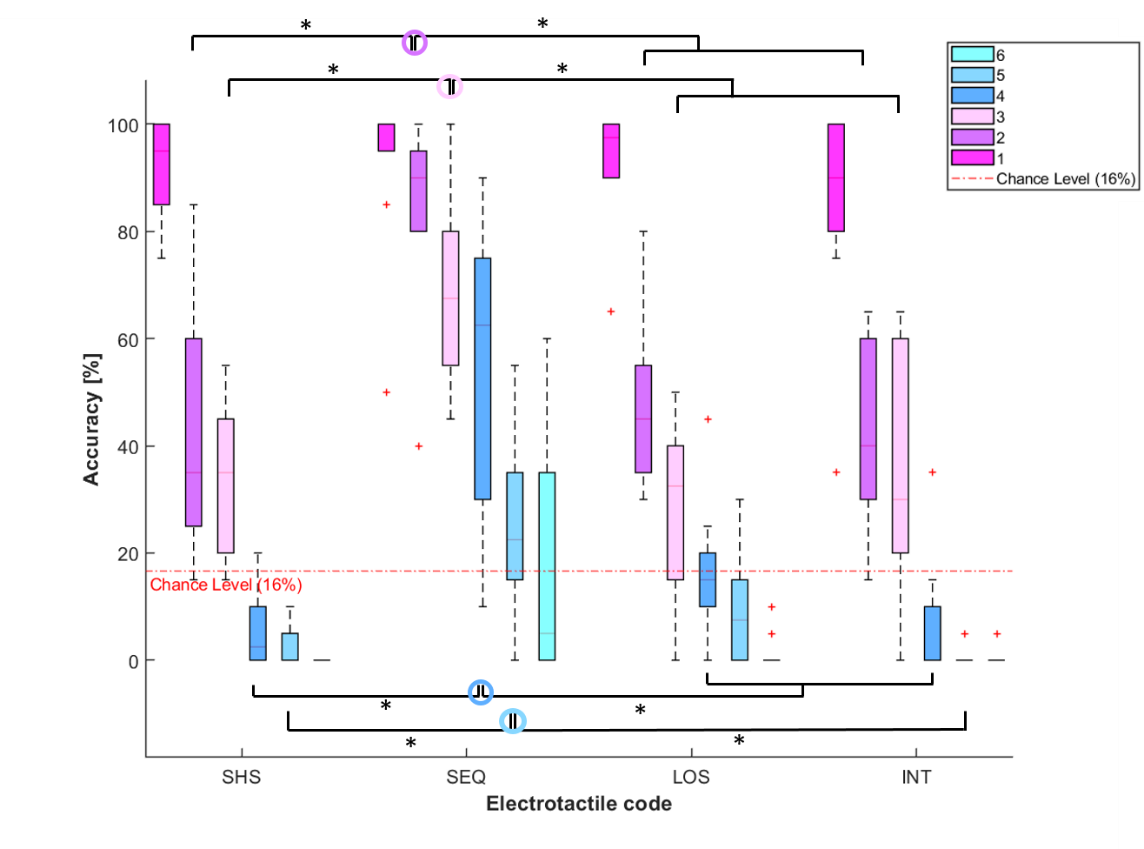


Figure 1. Boxplots, showing the median and 25 and 75 percentiles of the accuracy. Data is split for the four feedback codes (SHS, SEQ, LOS and INT). The different colors represents the different number of active electrodes (1 to 6). The dotted red line in the accuracy’s plot represent the chance level (16 %). Asterisks indicate statistical differences.


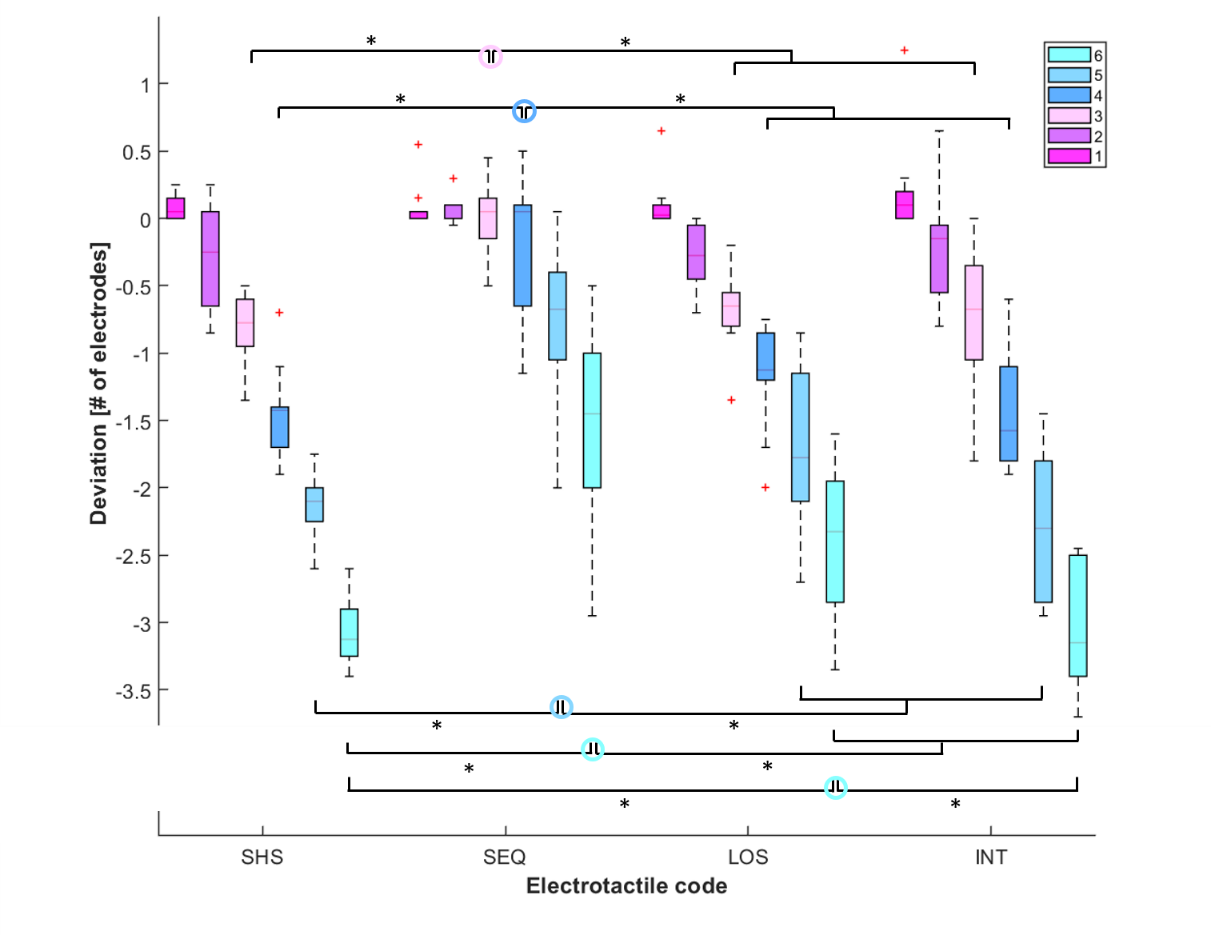


Figure 2. Boxplots, showing the median and 25 and 75 percentiles of the deviation. Data is split for the four feedback codes (SHS, SEQ, LOS and INT). The different colors represents the different number of active electrodes (1 to 6). Asterisks indicate statistical differences.
